# Amniotic fluid stem cells provide considerable advantages in epidermal regeneration: B7H4 creates a moderate inflammation microenvironment to promote wound repair

**DOI:** 10.1038/srep11560

**Published:** 2015-06-23

**Authors:** Qing Sun, Fang Li, Hong Li, Rui-Hua Chen, Yan-Zheng Gu, Ying Chen, Han-Si Liang, Xin-Ran You, Si-Si Ding, Ling Gao, Yun-Liang Wang, Ming-De Qin, Xue-Guang Zhang

**Affiliations:** 1 The Stem Cell and Biomedical Material Key Laboratory of Jiangsu Province (the State Key Laboratory Incubation Base), Soochow University, Suzhou, Jiangsu Province, P.R. China; 2 Jiangsu Institute of Clinical Immunology, The First Affiliated Hospital of Soochow University, Suzhou, Jiangsu Province, P.R. China; 3 Department of Immunology, School of Biology and Basic Medical Sciences, Soochow University, Suzhou, Jiangsu Province, P.R. China; 4 Department of Human Anatomy, Histology and Embryology, School of Biology and Basic Medical Sciences, Soochow University, Suzhou, Jiangsu Province, P.R. China; 5 Center for Reproduction and Genetics, Suzhou Hospital Affiliated to Nanjing Medical University, Suzhou, Jiangsu Province,P.R. China

## Abstract

The current treatments for severe skin injury all involve skin grafting. However, there is a worldwide shortage of donor skin tissue. In this study, we examined the advantages of using human amniotic fluid stem (hAFS) cells in skin wound healing. *In vitro*, hAFS cells differentiate into keratinocytes (termed hAFS-K). Like keratinocytes, hAFS-K cells express the markers K5, K14, K10 and involucrin; display typical cellular structure, including a tonofibril-rich cytoplasm; and construct a completely pluristratified epithelium in 3D culture. *In vivo*, in a mouse excisional wound model, GFP-positive hAFS cells participate in wound repair. Co-localization of GFP/K14 and GFP/K10 in the repaired epidermis demonstrated that hAFS cells can differentiate into keratinocytes. Real-time PCR results confirmed that hAFS cells can initiate and promote early-stage repair of skin damage. During wound repair, hAFS cells did not directly secrete repair-related factors, such as bFGF, VEGF, CXCL12, TGF-β1 and KGF, and provided a moderate inflammation reaction with lower expression of IL-1β, IL-6, TNF-α, Cox2 and Mac3. In hAFS cells, the negative co-stimulatory molecule B7H4 regulates low immunogenicity, which can provide a modest inflammatory reaction microenvironment for wound repair. Furthermore, with their uniquely high proliferation rate, hAFS cells offer a promising alternative for epidermal regeneration.

Several million people worldwide suffer from severe burns each year, and approximately 10% of burn patients die due to a lack of a suitable treatment. Currently, skin repair methods used to treat burns include autologous skin grafting^1^, allogeneic skin grafting^2^, and heterologous grafting. Problems with these approaches include a shortage of donor skin, immune rejection, the spread of disease^3^,^4^ and other shortcomings^2^, and there is therefore an urgent need to identify a high-quality skin substitute for clinical transplantation therapy. Human embryonic stem cells (hESCs) may offer a new approach to the treatment of skin injuries^5^, but they face ethical challenges^6^,^7^. Therefore, alternative seed cells such as adult stem cells may be a way forward for skin tissue engineering in regenerative medicine^8^,^9^. Foetal-derived stem cells represent one therapeutic possibility and can be routinely obtained from human amniotic fluid using backup cells from amniocentesis specimens that would otherwise be discarded^10^,^11^,^12^. These human amniotic fluid stem (hAFS) cells can be isolated using a number of protocols, including immunoselection with antibodies specific for c-Kit (CD117)^12^.

hAFS cells express both embryonic and adult stem cell markers and can be induced to differentiate into cell types derived from different germ layers, including cells of adipogenic, osteogenic, myogenic, endothelial, neuronal and hepatic lineages^10^,^11^,^13^,^14^. It has recently been reported that hAFS cells can form capillary-like networks^15^ and neuron-like cells^16^, but there is little information on their contribution to wound healing in skin. Flow cytometry showed that hAFS cells express the embryonic stem cell markers Oct-4, hTERT, SSEA-1, SSEA-4 and CD117^17^ but not SSEA-3^18^,^19^. These cells also express mesenchymal stem cell markers CD29, CD44, CD73, CD90 and CD105^10^,^20^,^21^,^22^,^23^, which are markers of the haematopoietic lineage, but are negative for CD45. hAFS cells also express both CD34 and CD133, markers of hematopoietic stem cells, suggesting that hAFS cells have the characteristics of embryonic stem cells^24^. In addition, hAFS cells exhibit low immunogenicity^25^.

Based on hAFS cells’ high proliferative potential, low immunogenicity and high differentiation potential, we hypothesize that hAFS may be a novel source for seed cells for epidermal regeneration with several advantages. In this study, we examined whether hAFS cells can differentiate into keratinocytes *in vitro* and contribute to epidermal regeneration *in vivo*. We conclude that B7H4 expression on hAFS cells is a key element for moderating inflammation and promoting wound repair. This finding may prove useful for future clinical applications of hAFS cells. We believe that hAFS cells can open a new field of stem cell study and provide a new source of seed cells for skin tissue engineering.

## Results

### Feasibility of the use of hAFS cells as seed cells for epidermal regeneration

**Figure 1:**
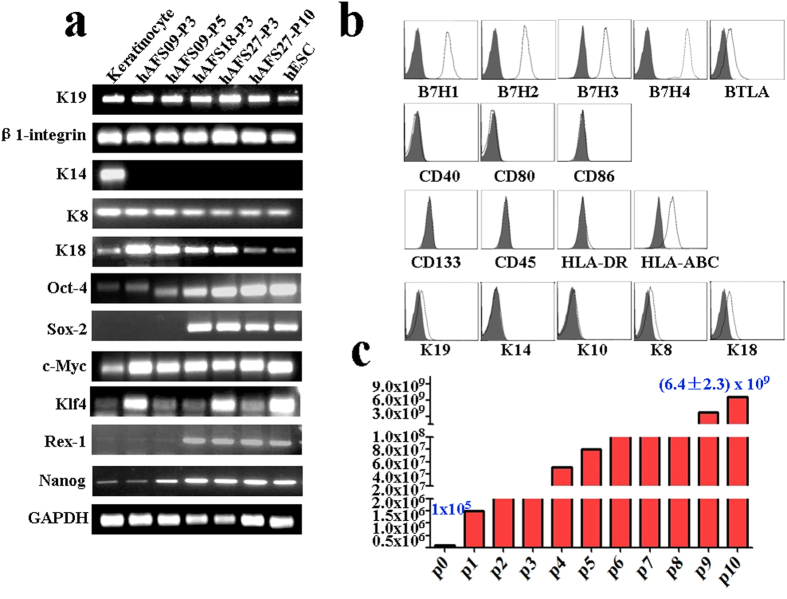
Biological characteristics of hAFS cells (**a**) mRNA expression of K19, β1-integrin, K14, K8, K18, Oct4, Sox2, c-Myc, Klf-4, Rex-1 and Nanog in keratinocytes, hAFS cell lines hAFS-09 (passages 3 and 5), hAFS-18 (passage 3), hAFS-27 (passages 3 and 10) and hESCs. Cropped gels were used, and the gels were run under the same experimental conditions. Full-length images are presented in Supplementary Figure S4. (**b**) Flow cytometry analysis of hAFS cell surface markers. hAFS cells (passage 5) were analysed after staining with PE-conjugated control isotype IgG (black peaks) or antibodies against the cell surface proteins B7H1, B7H2, B7H3, B7H4, BTLA, CD40, CD80, CD86, CD133, CD45, HLA-DR, HLA-ABC, K19, K14, K10, K8 and K18. (**c**) After sub-culturing hAFS ten times in succession, 1 × 10^5^ CD117-positive cells isolated from 5 ml of amniotic fluid reached an average of (6.4 ± 2.3) × 10^9^ cells (n = 4) in approximately one month.

RT-PCR analysis of three lines of hAFS cells with different passage numbers showed that they express K19 and β1-integrin (markers of epithelial stem cells) as well as K8 and K18 (markers of epithelium), indicating that hAFS cells have the potential to differentiate into epithelium, which represents one type of epithelial cell. In addition, hAFS cells expressed marker genes for embryonic stem cells, including Oct4, Sox2, c-Myc, Klf4, Rex-1 and Nanog (Fig. 1a). After 10 passages, subcultures of hAFS cells maintained a gene expression pattern similar to that of early-passage cells.

Flow cytometry was carried out to characterize the expression of immune-related markers in hAFS cells. We found that hAFS cells were negative for the positive co-stimulatory molecules CD40, CD80 and CD86 but showed strong expression of the negative co-stimulatory molecules B7H1, B7H2, B7H3, B7H4 and BTLA. This finding suggests that hAFS cells may be involved in inhibiting lymphocyte activation and suppressing inflammatory responses. Cultures of hAFS cells were negative for CD45, CD133, and HLA-DR but positive for HLA-ABC. Flow cytometry results confirmed that hAFS cells were positive for K19, K8, and K18 but negative for K14 and K10 (Fig. 1b).

To test the proliferation of hAFS, 1 × 10^5^ CD117-positive cells were isolated from 5 ml amniotic fluid after one month and amplified to (6.4 ± 2.3) x 10^9^ cells after being sub-cultured ten times, during which time they maintained the characteristics of low-passage-number hAFS cells. This behaviour demonstrated that hAFS cells have strong proliferative potential (Fig. 1c).

### hAFS cells are able to differentiate into keratinocytes *in vitro*

**Figure 2:**
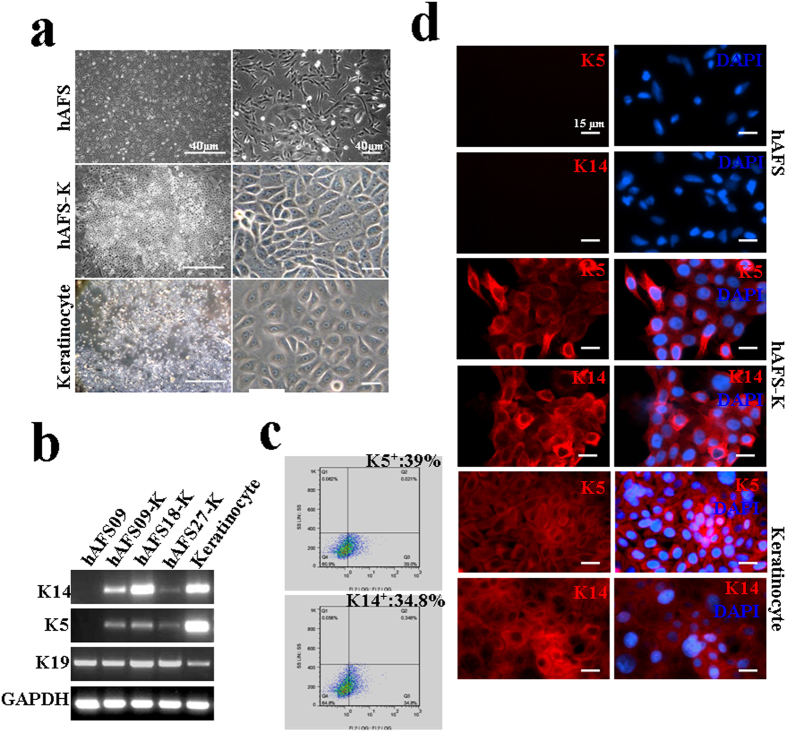
Differentiation of hAFS cells and characterization of hAFS cell-derived keratinocytes (**a**) The morphology of hAFS cells, hAFS cell-derived keratinocytes (hAFS-K) and human keratinocytes. After induction, hAFS-K exhibited typical pavementous epithelial morphology and spontaneously formed colonies similar to keratinocytes. (**b**) mRNA expression of K5, K14, K19 in hAFS-K. hAFS cells were used as a negative control and keratinocytes as a positive control. Cropped gels were used, and the gels were run under the same experimental conditions. Full-length images are presented in Supplementary Figure S5. (**c**) hAFS-K (n = 4) were analysed by flow cytometry after staining with PE-conjugated control isotype IgG (grey peaks) or antibodies against cell surface proteins K5 and K14, showing the differentiation rates to be (40 ± 1.24)% and (35 ± 0.36)%, respectively. (**d**) Immunofluorescence staining of K5 and K14 (red) in hAFS cells, hAFS-K and keratinocytes. Nuclei (blue) were stained with DAPI. hAFS cells were used as the negative control and keratinocytes as the positive control.

To stimulate differentiation into keratinocytes, hAFS cells were grown in inducing medium for approximately 30 days and then maintained in KGM2 without serum. After the third passage, colonies of cells formed spontaneously with typical pavementous epithelial morphology; we named these cells hAFS-K (keratinocytes derived from hAFS cells). The phenotypic characteristics of hAFS-K were similar to those of human primary keratinocytes but clearly distinct from those of hAFS cells (Fig. 2a). RT-PCR results identified hAFS-K as K5 and K14 positive, like basal keratinocytes, with comparable gene expression levels in three independent cell lines (Fig. 2b). Flow cytometry analysis of K5- and K14-positive hAFS-K cells showed the differentiation rate to be (40 ± 1.24% and 35 ± 0.36%, respectively (Fig. 2c), and immunostaining confirmed that the cells express the K5 and K14 proteins (Fig. 2d). Taken together, these results demonstrate that hAFS-K cells have similar biological characteristics to keratinocytes.

### Keratinocytes derived from hAFS cells form a complete pluristratified epithelium

**Figure 3:**
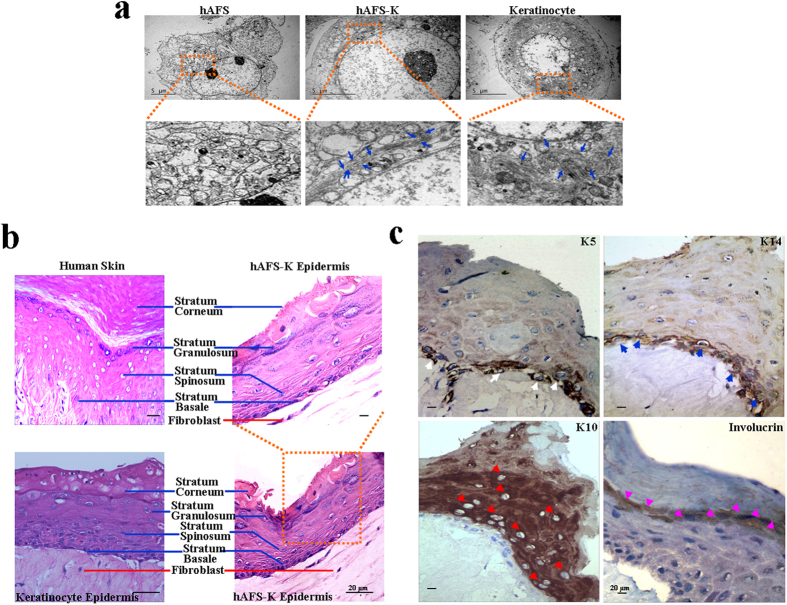
Ultrastructural characteristics of hAFS cells and differentiation in 3D culture (**a**) Transmission electron microscope images of hAFS cells, hAFS-K and mature keratinocytes. Insets show tonofibrils in hAFS-K and keratinocytes (blue arrows), which are missing in hAFS cells. (**b**) H&E images of normal human skin and the epidermis constructed by hAFS-K demonstrate a well-organized, multilayer epithelium that contains all morphological layers (blue lines) and a collagen matrix in which resident fibroblasts (red line) play the role of the dermis. An epidermis constructed by keratinocytes was used as a positive control. (**c**) Immunohistochemistry of an organotypic epidermis constructed by hAFS-K showing a normal distribution of epidermis markers: K5 in the basal layer (white arrow); K14 in the basal layer (blue arrow); the marker of mature keratinocytes, K10 (red arrow); and Involucrin (pink arrow) in the upper layers.

To further examine the biological function of hAFS-K, we compared the cellular structure of hAFS cells, hAFS-K cells and mature keratinocytes. Although hAFS cells have an expanded endoplasmic reticulum, a clearly visible nucleolus, and a high ratio of nucleus to cytoplasm (consistent with a high proliferation rate), they lack tonofibrils (organelles typical of keratinocytes). In contrast, mature keratinocytes contain multi-filamentary structures and conspicuous tonofibrils and show a low ratio of nucleus to cytoplasm. hAFS-K cells display features of keratinocyte precursor cells, with tonofibrils distributed in the cytoplasm, but these cells also have a prominent nucleolus and a high nucleus-to-cytoplasm ratio (Fig. 3a).

hAFS-K cells resemble normal keratinocytes phenotypically and functionally, as demonstrated in a 3D tissue culture system that features a collagen matrix in which resident fibroblasts play the role of the dermis. After 8 days of air-liquid differentiation, H&E staining of organotypic cultures of hAFS-K showed a pluristratified epithelium with a basal layer, a stratum spinosum, a stratum granulosum containing keratohyalin granules, and a stratum corneum observed as superposed layers of dead squamous enucleated cells. Epidermis derived from keratinocytes was used as a positive control (Fig. 3b). Differentiation markers were expressed normally and localized in layers. Immunohistochemistry showed that K14 and K5 were present in the basal compartment but to a much lesser extent in the suprabasal layers. A reciprocal pattern was found for K10, for involucrin, and for markers of mature keratinocytes in suprabasal layers, which was present only in layers overlying the basal layer (Fig. 3c). In this serum-free co-culture model, fibroblasts provide all of the necessary growth factors and extracellular matrix proteins needed to support keratinocyte differentiation^26^, allowing hAFS-K cells that require fibroblasts or fibroblast products to grow and function properly. In conclusion, hAFS-K cells can form a completely pluristratified epithelium in 3D air-liquid tissue cultures.

### hAFS cells differentiate into keratinocytes *in vivo* during skin damage repair

**Figure 4:**
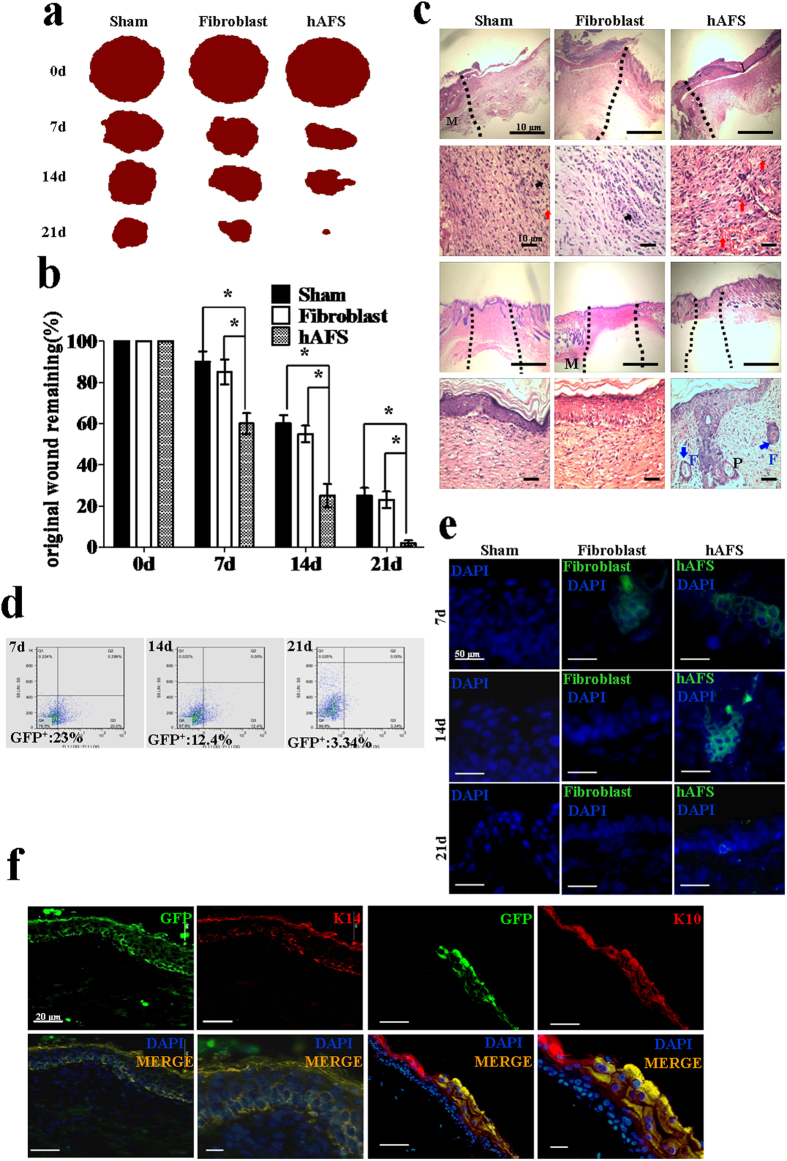
GFP-positive hAFS cells directly promote and contribute to wound healing *in vivo* (**a**) Representative photographs of the wounds with GFP-positive hAFS cells, fibroblasts or sham cells at day 0, day 7, day 14 and day 21 are shown. (**b**) Measurement of wounds remaining in the sham, fibroblast, and hAFS cell groups in BALB/c mice. Analysis of variance (ANOVA) versus sham or fibroblast groups. The wounds were measured using the UTHSCSA ImageTool; *p < 0.01. (**c**) Histological analysis of wounds on BALB/c mice in the sham, fibroblast and hAFS groups at days 4 and 14 (H&E stain). At day 4, the wound edge is indicated by the black dashed lines. The infiltrated inflammatory cells (black arrowheads) and the capillary-like structures (red arrowheads) are indicated in the granulation tissue. At day 14, the repaired skin area is indicated by the black dashed lines, and newly formed hair follicles (blue arrowheads) are also shown. Abbreviations: M, muscle; F, hair follicle; P, papillae. (**d**) Flow cytometry of GFP-positive cells in the wound at 7, 14 and 21 days after wounding. (**e**) Immunofluorescence analysis of the presence of GFP-positive hAFS cells and fibroblasts in the wound at 7, 14 and 21 days. (**f**) Tissue sections from hAFS cell-treated wounds at day 7 were stained with K14 and K10 antibodies in red. The co-localization of K14 with GFP and K10 with GFP in the merged images appears orange.

A GFP reporter gene was introduced into hAFS cells and fibroblasts using a lentiviral vector to enable the cells to be traced in the subsequent experiments. Flow cytometry and immunofluorescence showed that almost 100% of hAFS cells and fibroblasts were GFP positive (Suppl. Fig. S1a). We used an excision wound model in BALB/c mice with a ring to reduce self-repair, as previously described^27^. GFP-positive hAFS cells were injected into the wound bed. Over time, wounds treated with hAFS cells exhibited accelerated wound closure when compared to fibroblast-treated wounds or sham groups (Fig. 4a and Suppl. Fig. S1b). At day 7, there was more wound healing in hAFS cell-treated mice than the fibroblast and sham groups. At day 21, the wounds in hAFS cell-treated mice (n = 12) achieved almost complete wound closure, whereas no completely closed wounds were observed in the fibroblast-treated (n = 8) or sham group (n = 7) mice. These results show that hAFS cells can quickly and efficiently promote wound healing (Fig. 4b).

Histological analysis of wounds in BALB/c mice at day 14 revealed enhanced re-epithelialization in hAFS-treated wounds when compared with fibroblast-treated wounds or the sham group. Analysis of wounds at day 4 indicated that granulation tissue in hAFS-treated wounds contained more fibroblasts and capillary-like structures but fewer infiltrated inflammatory cells (Fig. 4c). According to the analysis of wounds at day 14, hAFS-treated wounds appeared to enhance the formation of skin appendage-like structures (Fig. 4c), suggesting enhanced cutaneous regeneration.

To determine the proportion of GFP-positive cells engrafted into the wounded skin at different times, we excised the entire wound along with a small amount of the surrounding skin and dispersed it into a single-cell suspension. Flow cytometry analysis showed that a large number of GFP-positive cells were present in hAFS cell-treated wounds at day 7, at (25 ± 2.42)%, with this proportion declining to (12 ± 1.56)% at day 14 and to (3 ± 0.54)% at day 21 (Fig. 4d). Immunofluorescence analysis revealed the existence of the GFP-positive hAFS cells and fibroblast in the wound at 7, 14 and 21 days. The number of cells (including hAFS cells and fibroblasts) in the wound declined over time (Fig. 4e). The reasons for the decline the in numbers of implanted hAFS cells over time are not fully understood. It is likely that, with progression of wound healing, the concentrations of cytokines that are favourable for hAFS cell survival and engraftment decrease^28^,^29^,^30^. On day 7, cells that were positive for both GFP and K14 were located in the basal layer of the skin, whereas GFP and K10 double-positive cells were detected in the upper layers, showing that keratinocytes derived from GFP-positive hAFS had undergone maturation (Fig. 4f). These data demonstrate that hAFS cells have the capacity to differentiate into keratinocytes *in vivo*.

### hAFS cells orchestrate local epidermal regeneration

**Figure 5:**
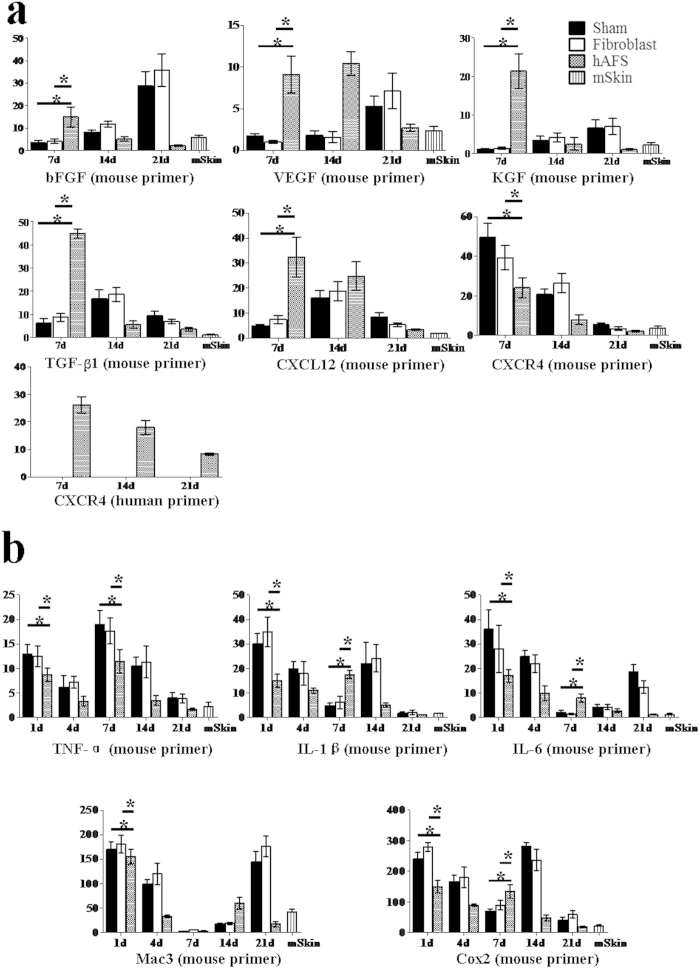
hAFS cells indirectly promote wound healing ***in vivo*** (**a**) Real-time PCR analysis of mRNA expression of repair-related factors (bFGF, VEGF, TGF-β1, KGF, CXCL12, and CXCR4) at days 7, 14, 21 in hAFS cell-treated wounds, the fibroblast group and a sham control group. (**b**) Real-time PCR analysis of mRNA expression of inflammatory factors (TNF-α, Cox2, Mac3, IL-6, IL-1β) at days 1, 4, 7, 14 and 21 in hAFS cell-treated wounds in the fibroblast and sham control groups. Both mouse and human sequences were quantified in each case. There was no expression of human bFGF, VEGF, CXCL12, TGF-β1, KGF, TNF-α, Cox2, Mac3, IL-6, or IL-1β in either hAFS-, fibroblast- or sham-treated wounds. The data are presented as the mean ± SD of three independent experiments. Analysis was performed with GraphPad Prism; *p < 0.05.

To explore the role of hAFS cells in skin repair, 11 mouse and human factors including repair-related and inflammatory factors were analysed with species-specific primers. There was no expression of human bFGF, VEGF, CXCL12, TGF-β1, KGF, TNF-α, Il-1β, Il-6, Cox2 or Mac3 in hAFS-treated, fibroblast groups or sham group wounds. As shown in Fig. 5a, the mRNA levels of mouse bFGF were highest in the hAFS cell group at day 7 and much higher than in the fibroblast and sham group but then reduced gradually through days 14 and 21. This pattern indicates that hAFS cells initiate the expression of bFGF at an early stage. In contrast, the expression of mouse bFGF in the fibroblast and sham groups reached a maximum at day 21 before the repair was complete. A similar pattern was observed for mouse VEGF, CXCL12, TGF-β1 and KGF at day 7 (Fig. 5a). Both mouse and human CXCR4 were detected, suggesting that both mouse and human AFS cells were involved in wound repair. During the course of wound healing, both mouse and human CXCR4 mRNA levels decreased, possibly because the stem cells leave the wound site after repair (Fig. 5a). Thus, these data suggest that, although no human bFGF, VEGF, KGF, TGF-β1 or CXCL12 were detected, hAFS cells were involved in the initiation of skin repair in the mouse model.

hAFS treatment stimulated the expression of wound healing factors, which indicates that hAFS cells are able to indirectly initiate vasculogenesis and re-epithelialization as part of the wound repair process. Because of the high expression of inflammatory cytokines in the early stages of wound repair, we also tested samples at 1 and 4 days for inflammatory cytokines (Fig. 5b). The expression of TNF-α in the fibroblast and sham groups was much higher than in the hAFS-treated group on the first day and decreased at day 4. At day 7, the expression of TNF-α reached a second peak. In contrast, the expression of TNF-α in the hAFS-treated group was moderate. By the end of wound healing, the expression of TNF-α was the same as its expression in normal mouse skin. A similar pattern was observed in the expression of other factors (IL-1β, IL-6, Cox2 and Mac3). Therefore, hAFS can provide a moderate inflammation reaction microenvironment, which effectively promotes the wound repair process (Fig. 5b).

### The mechanism of hAFS cells’ effective promotion of wound repair by creating a moderate inflammatory microenvironment

**Figure 6:**
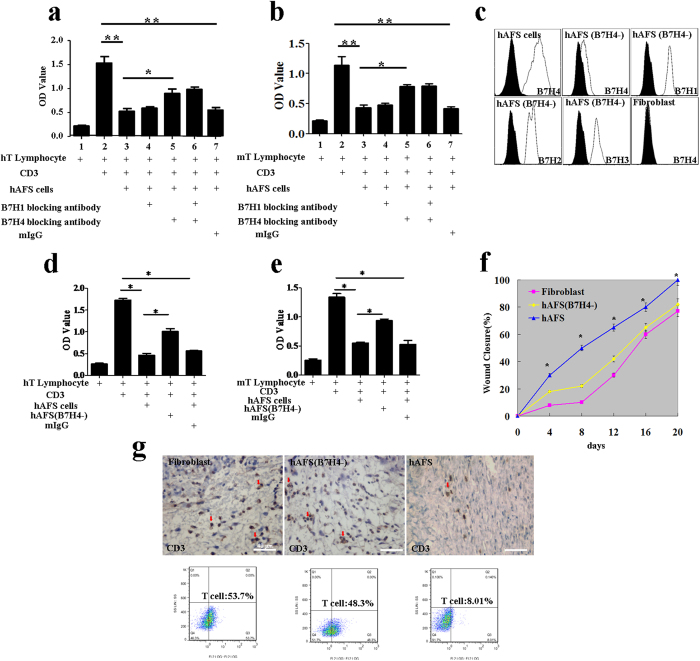
The role of the B7H4 molecule in epidermal regeneration (**a**) Analysis of the inhibitory effect of hAFS cells on human lymphocytes and the effect of the B7 family blocking antibody on hAFS inhibitory ability. The data are presented as the mean ± SD of three independent experiments. Analysis was performed with GraphPad Prism; *p < 0.05, **p < 0.01. (**b**) Analysis of the inhibitory effect of hAFS cells on mouse lymphocytes. The effect of the B7 family blocking antibody on hAFS cells’ inhibitory ability. The data are presented as the mean ± SD of three independent experiments. Analysis was performed with GraphPad Prism; *p < 0.05; **p < 0.01. (**c**) hAFS cells, fibroblasts and B7H4-downregulated hAFS cells were analysed by flow cytometry after staining with PE-conjugated control isotype IgG (black peaks) or antibodies against the cell surface proteins B7H4. B7H4-downregulated hAFS cells were also analysed by flow cytometry after staining with PE-conjugated control isotype IgG (black peaks) or antibodies against the cell surface proteins B7H1, B7H2 and B7H3. (**d**) Analysis of the inhibitory effect of B7H4-downregulated hAFS cells on human lymphocytes. (**e**) Analysis of the inhibitory effect of B7H4-downregulated hAFS cells on mouse lymphocytes. (**f**) Measurement of wound closure in the B7H4-downregulated hAFS, fibroblast, and hAFS cell groups in BALB/c mice. Analysis of variance (ANOVA) of wounds measured using the UTHSCSA ImageTool; *p < 0.01. (**g**) Infiltration of CD3+ T cells (red arrow) into the granulation tissue of BALB/c mice in the fibroblast, B7H4-downregulated hAFS and hAFS groups at day 4. When compared with the hAFS group, more infiltrated CD3+ T cells are visible in the fibroblast and B7H4-downregulated hAFS groups.

A low immune response can result in moderate inflammation in wound repair. To identify the mechanism of hAFS-mediated low immunogenicity, CD3 was used to stimulate the activation of human and mouse T lymphocytes. Mitomycin-treated hAFS cells can inhibit the activity of human and mouse T lymphocytes. We hypothesized that members of the B7 family play an important role; therefore, we downregulated B7H1 and B7H4 expression using specific blocking antibodies. The hAFS B7H4-blocking antibody can significantly inhibit the suppression of human and mouse T lymphocytes by hAFS cells without B7H1 effects or B7H1-B7H4 combined effects (Fig. 6a,b and Suppl. Fig. S2a, S2b). T-cell proliferation assays indicate that the contribution of B7H4 is approximately 50% of the T-cell suppression function of hAFS cells. These results suggest that B7H4 is an important molecule facilitating the low immunogenicity of hAFS cells.

To further explore the effects of hAFS B7H4 expression on low immune responses and moderate inflammation reaction during wound repair, B7H4 expression was downregulated in hAFS cells by using a lentiviral vector that carries an interfering RNA for B7H4. The expression of B7H4 in the hAFS cells was almost 100%, whereas fibroblasts did not express B7H4. In the B7H4 downregulated hAFS cells, mRNA levels were reduced by approximately 20%. The RNAi lentivirus for B7H4 did not affect normal expressions of B7H1, B7H2 and B7H3 in B7H4 downregulated hAFS cells (Fig. 6c). Furthermore, B7H4 downregulation in hAFS cells relieved the suppression of both the human and mouse T-cell activation (Fig. 6d,e), which confirmed that B7H4 downregulation in hAFS cells relieves the low immunogenicity. To evaluate the effect of the expression of B7H4 in hAFS cells on the wound repair process, B7H4-downregulated hAFS cells and fibroblasts were separately injected into the wound bed. The hAFS cell group repaired the skin damage faster and more effectively than the B7H4-downregulated and fibroblast groups (n = 8) (Fig. 6f). To demonstrate the B7H4-mediated immune reaction, we identified the number of infiltrated CD3^+^ T cells in the granulation tissue in the hAFS groups at day 4. The fibroblast and B7H4-downregulated groups had more visible infiltrated CD3^+^ T cells than the hAFS group. The percentages of T cells in the fibroblast group, the B7H4 downregulated hAFS group and the hAFS cells group are (55 ± 3.2)%, (49 ± 4.23)% and (9.4 ± 2.2)%, respectively (Fig. 6g). B7H4-downregulated hAFS cells’ relief of accelerated repair was further confirmed by an analysis of repair-related factors and inflammatory factors. The expression of repair-related factors (bFGF, VEGF, TGF-β1, KGF) in hAFS cells was higher than in the B7H4-downregulated hAFS cells and fibroblasts by day 7, whereas the B7H4-downregulated hAFS cells and fibroblast groups reached the highest expression of these factors at days 14 or 21 (Suppl. Fig. S3a). The expression of inflammatory factors (IL-1beta, IL-6, TNF-α, Cox2 and Mac3) in hAFS cells was slightly higher than in B7H4-downregulated hAFS cells at day 7. In the later stages, the expression of inflammatory factors in the hAFS group was more moderate than in the other two groups (Suppl. Fig. S3b). All of these phenomena confirm that B7H4 can regulate low immunogenicity and lead hAFS cells to create a mild repair microenvironment to effectively promote wound repair.

## Discussion

Alternatives to grafting for skin wound repair are urgently needed due to a global shortage of donor skin tissue. We assessed the potential of hAFS cells as a novel therapeutic option. hAFS cells can differentiate into keratinocyte precursors *in vitro*, as evidenced by the expression of keratinocyte markers (K5 and K14). hAFS cells mature further with the expression of K10, involucrin and the formation of typical organelles (tonofibril-rich cellular structure). These cells can also construct a completely pluristratified epithelium in 3D air-liquid interface tissue culture. In our experiment, we used the cytokine BMP4, which has been shown to inhibit neural induction and to maintain epithelial commitment^31^. This activity may occur because BMP4 and BMP cytokine signalling plays a key role in early ectodermal fate choices during development in multiple species^32^,^33^,^34^. BMP4 has also been shown to induce keratinocyte differentiation of mESCs^35^ through the Smad pathway^34^. hAFS cells are similar to hESC to a certain extent. We also tested the RA in our experiment at several different concentrations, but the hAFS cells were sensitive to RA, and RA treatment caused cell death.

The biological characteristics and origin of hAFS cells suggest they have unique potential for epidermal regeneration. It has been reported that amniotic fluid contains multiple cell types derived from the developing foetus, including cells from foetal skin, the respiratory system, and the urinary and gastrointestinal tracts^36^,^37^. If the hAFS cell population includes cells from foetal skin, this could be a distinct advantage for seeding skin in wound repair. It has been reported that hAFS cells can give rise to ectodermal cells, including neuron-like cells^16^, complementing our finding of the expression of epithelial stem cell marker genes such as K19 and β1-integrin, indicating an epithelial origin. The simultaneous expression of marker genes such as K8 and K18 for epithelium, as well as the stem cell markers Oct4, Sox2, c-Myc, Klf4, Rex-1 and Nanog, make hAFS cells novel and attractive candidates for use in epithelial regeneration.

We also examined the ability of hAFS cells to repair skin injury *in vivo* in a mouse excision wound model. After the introduction of GFP-positive hAFS cells into the wound bed, immunofluorescence showed the co-localization of GFP/K14 and GFP/K10 in the epidermis, proving that hAFS cells can differentiate into keratinocytes and directly participate in damage repair in the wound (i.e., they have a direct effect). Furthermore, in the wound, hAFS cells can initiate repair by promoting the expression of bFGF, VEGF, TGF-β1, KGF and CXCL12/CXCR4. During wound repair, it was intriguing to note that hAFS cells themselves did not directly secrete repair-related factors such as bFGF, VEGF, TGF-β1, KGF and CXCL12, suggesting that hAFS cells may promote wound healing indirectly. That is to say, hAFS cells may not only differentiate into keratinocytes directly in the early stage of repair but also have a substantial but indirect effect throughout the repair process. The results are consistent with previous works^38^.

Low immunogenicity is another property of hAFS cells^25^,^39^. Emily^25^ and his team hypothesized that cells in amniotic fluid may have an immunoprivileged status, as foetal cells possess mechanisms to avoid destruction by the maternal immune system during development. In this study, we found that hAFS cells did not express the positive co-stimulatory molecules CD40, CD80 and CD86 but did express the negative co-stimulatory molecules B7H1, B7H2, B7H3, B7H4 and BTLA, consistent with low immunogenicity. Unselected mesenchymal stromal cells from amniotic fluid are known to inhibit lymphocyte proliferation *in vitro*^28^. Our findings suggest that hAFS can inhibit the activity of human and mouse T lymphocytes and that the negative co-stimulatory molecule B7H4 is an important molecule for mediating the low immunogenicity of hAFS. These data indicate that hAFS cells may be an attractive candidate for eventual therapeutic applications in skin wounds.

Inflammation is a defence response to potentially damaging stimuli, but it is a double-edged sword with regard to wound healing. Typically, inflammation is helpful and is an automatic defence response by the body. An excessive level of inflammation can be harmful, for example, via its effects on the body’s tissues, when attacks occur in transplanted tissue. hAFS cell treatment elicited a moderate inflammatory response with a lower expression of IL-1β, IL-6, TNF-α, Cox2 and Mac3 compared to fibroblast and sham groups, resulting in a better microenvironment for wound repair. However, the downregulation of B7H4 led hAFS cells to lose these advantages during wound repair. The results indicate that B7H4 is an important factor for the contribution of hFAS cells to wound repair.

Finally, hAFS cells are rich-sample resources, are easily obtained, do not form tumours after transplantation in mice^40^ and are free of ethical challenges. It has been reported that hAFS cells can be amplified over 300-fold in the laboratory, suggesting that large quantities could be produced^41^. Based on our *in vivo* epidermal regeneration study, 5 × 10^6^ hAFS cells can repair a mouse skin wound with a diameter of 1 cm. Thus, if (6.4 ± 2.3) × 10^9^ hAFS cells can be obtained after *in vitro* culture, there are enough cells for clinical treatment of skin injuries. Taken together, the present study identifies hAFS cells as a new source of keratinocytes that are able to form an epidermis, making these cells a potentially vital resource for patients requiring urgent treatment of a large area of damaged skin.

## Methods

### Ethics statement

All methods were carried out in accordance with the approved guidelines. All experimental protocols were approved by Soochow University. In this study, hAFS samples were collected with the written consent of subjects and the written approval of the ethical review board of the Suzhou Hospital, affiliated with Nanjing Medical and Soochow University. Copies of the written consent provided by the subjects along the written approval from the review board were kept in the hospital ethical review board office. All experimental procedures using hAFS samples in this study were reviewed and approved by the ethics committee. Mice used in the present study were handled in strict accordance with best animal practices. All experimental procedures using mice in this study were reviewed and approved by the ethical review board of Soochow University.

### Isolation and culture of hAFS cells

Samples of amniotic fluid (AF) were obtained from Suzhou Hospital Affiliated with Nanjing Medical University following routine amniocentesis carried out on pregnant women after 19-22 weeks of gestation. All procedures were performed following the guidelines established by Suzhou Hospital Affiliated with Nanjing Medical University Ethics Boards. Written consent was obtained from each woman after informing her that the amniotic fluid would be used for both genetic analysis and research purposes. After amniocentesis, immunoselection with an antibody specific for human c-Kit (CD117) was used to isolate AFS cells^12^. The cells were isolated from each AF sample and then plated into a 10 cm culture dish (Corning) and expanded. The total cell count in 5 ml of amniotic fluid amounted to approximately 1 × 10^6^, of which approximately 1 × 10^4^ were hAFS cells. Approximately 95% of the non-adherent cells were removed after 24 h, and the culture media was replaced every day. Cells were passaged by trypsinization (0.25% trypsin, 0.1% EDTA) and expanded serially with a split ratio of 1:3 at 70% confluence in 2 or 3 days. Cultures of hAFS cells were maintained in a humidified incubator under 5% CO_2_ at 37 °C.

### Preparation of PBMCs and human T cells

Peripheral blood mononuclear cells (PBMCs) were isolated from blood with the Ficoll-Biocoll Separation Solution as previously described^42^. Blood from healthy donors was provided by the Suzhou Red Cross Blood Center. Human T cells were purified from PBMCs by negative selection using the MACS CD3 Isolation Kit (Miltenyi Biotec GmbH, Bergisch Gladbach, Germany). The purity of the isolated human T cells was >95%. The cells were cultured in RPMI-1640 medium (Gibco).

### Isolation of mouse lymphocytes

Splenocytes and thymocytes were obtained using PBS washes of the respective organs. Tissue was disaggregated by passing the digest through a 70-μm nylon mesh cell strainer (BD Falcon) in RPMI containing 10% FBS to obtain a single-cell suspension. Erythrocytes were eliminated by incubation for 5 min at room temperature in ACK lysis buffer. The cells were stained with anti-mouse CD3 plus isotype IgG control antibodies. The purity of these T-cell populations was >95% when analysed.

### T-cell proliferation assays

To assess the potential effect of hAFS cells on T-cell proliferation induced by CD3, hAFS cells were treated with mitomycin C (10 μg/ml) (Sigma–Aldrich) for 3 h at 37 °C and were washed with PBS containing 10% FBS. The inactivated hAFS cells were then added to proliferation assay cultures. For the CD3-stimulated proliferation assay, hAFS cells were plated in triplicate into 96-well plates and CD3 (10 μg/ml) was added. The ratio of T cells to hAFS cells was usually 5:1. The cultures were kept at 37 °C in an atmosphere of 5% CO_2_. Cell proliferation was measured at 72 h after incubation using the cell counting kit-8 (CCK8) assay. The proliferation assays were performed in triplicate.

### Blocking experiments

Blocking experiments were performed to determine the possible roles of B7H1 and B7H4 in hAFS-mediated immunoregulation. Neutralizing monoclonal antibodies (mAb) against human B7H1 and B7H4 (10 μg/ml) were added to CD3-induced proliferation assays. The same concentration of mouse IgG was used as an isotype control. Blocking studies with these mAbs and an isotype control were also performed for early T-cell activation studies. Mouse anti-human B7H1 mAb (clone: 2H11)^43^ and B7H4 mAb (clone: 3C8) were generated in our laboratory.

### Keratinocyte differentiation protocol

Third-passage hAFS cells were seeded in 6-well plates at a density of 2 × 10^4^ cells per well. In the experimental group, inducing medium (keratinocyte basal medium-2 (Lonza, Walkersville, MD) supplemented with 10% FBS, 10 ng/ml recombinant human EGF (R&D Systems, Minneapolis, MN), 5 μg/ml insulin (Sigma-Aldrich), 0.5 μg/ml hydrocortisone (Sigma-Aldrich), 10^−10^ M cholera toxin (Sigma-Aldrich), 1.37 ng/ml triiodothyronine (Sigma-Aldrich), 24 μg/ml adenine (Sigma-Aldrich), 0.5 nM human recombinant BMP4 (R&D Systems), 50 μg/ml ascorbic acid (Sigma-Aldrich) and keratinocyte-conditioned medium at a ratio of 1:1) was used. The original medium was used for the control group. After 30 days, the cells were grown in KGM2 medium (Lonza) without serum.

### RNA isolation and reverse transcription-polymerase chain reaction

Total RNA was isolated from hAFS cells, hAFS-K and keratinocytes using the RNeasy Mini extraction kit (Qiagen, Courtaboeuf, France) according to the manufacturer’s protocol. A total of 1 μg RNA was used for reverse transcription. Primers are shown in Table 1. For real-time PCR assays, the primer mixes were loaded in duplicate wells in 96-well plates and then performed after the addition of SYBR Green PCR Master Mix and 1 μg (final) cDNA. As an internal control, levels of GAPDH were quantified in parallel with target genes. Normalization and fold changes were calculated using the 2^−△△Ct^ method^31^. Primers are shown in Table 2.

### Three-dimensional air-liquid interface tissue culture

Collagen I from rat tail (Gibco), populated with fibroblasts that were isolated from foreskins, was used for fabricating successive layers of acellular and cellular collagen on a polycarbonate membrane. The collagen I was prepared as previously described^44^. After approximately 7 days, 1 × 10^5^ hAFS-K cells were added to the centre of the collagen gel and incubated for 1 h at 37 °C to allow the hAFS-K cells to fully adhere. Cells were exposed to the air-liquid interface for 8 days in Epilife medium (Gibco).

### Transmission electron microscopy

Samples were fixed for 4 h in 4% glutaraldehyde in 0.1 M cacodylate buffer pH 7.2 at 4 °C and post-fixed for 1 h in 1% osmium tetroxide at 4 °C in the dark. Samples were then dehydrated in a graded acetone series and embedded in Epon resin 812 (TAAB). Ultrathin sections (60 nm) were mounted on collodion carbon-coated copper grids, contrasted using uranyl acetate and lead citrate and examined at 60 kV with a transmission electron microscope (Philips CM12). Images were collected with a 4000 M-T1-GE-AMT detector (DVC Co., Austin, TX) and processed with Image J software (National Institutes of Health, Bethesda, MD).

### GFP-positive hAFS cells, B7H4 downregulated hAFS cells and GFP-positive fibroblasts

A lentivirus containing the GFP gene (LV-EGFP) was obtained from Sidansai Stem Cell Technology Co. at a titre of 6.5 × 10^6^ IU/ml. Lentivirus containing the GFP gene and interfering RNA for B7H4 downregulation were constructed using the same vector. We seeded 2 × 10^5^ cells and then added 1.3 × 10^6^ IU/ml of lentivirus with 10 mg/ml Polybrene. After 24 h, the medium was changed. When the hAFS cells and fibroblasts isolated from foreskins were nearly 80% confluent, GFP-positive cells were detected by FACS.

### Wound-healing model and GFP-positive hAFS cell transplantation

BALB/c male mice (25–30 g) were obtained from the Chinese Academy of Sciences and were randomly divided into three groups. An injury model was generated according to established methods^45^. Briefly, after anaesthesia, a full-thickness skin wound (1 cm diameter and approximately 1 mm depth) was made on the back of the mouse. The wound received a total of 5 × 10^6^ GFP-positive hAFS cells or fibroblasts at six injection points at the edge of the wound with a 120 μl cell suspension at each point. We covered the wound with gauze, packed the wound and then housed the mice individually. The wounds were measured using the UTHSCSA ImageTool. The animals were observed every day, and there were no unusual or allergic reactions.

### Flow cytometry analysis

Cells were detached from 6-well plates using trypsin and were re-suspended in phosphate-buffered saline (PBS) containing 1% bovine serum albumin (BSA). The cells were incubated at 4 °C for 1 h with primary antibodies specific for B7H1, B7H2, B7H3, B7H4, CD40, CD80, CD86 and BTLA (BD Pharmingen, San Diego, USA); specific for K19, K10 and K14 (Santa Cruz Biotechnology, Santa Cruz, CA, USA); or specific for K8 and K18 (Chemicon, USA). Negative controls were performed by omitting the primary antibodies. Cells were re-suspended in PBS containing phycoerythrin (PE)-labelled goat anti-mouse IgG secondary antibodies (Santa Cruz Biotechnology). For detection of GFP-positive cells in the wounded skin, excised wounds, together with a small amount of surrounding skin, were dispersed into single-cell suspensions as previously described^46^. Cells from the wounds of the sham group were used as negative controls. Ten thousand events were analysed using a FACSCalibur flow cytometer (Becton-Dickinson, Franklin Lakes, NJ).

### Histological and immunochemical analyses

Cells were fixed in 4% paraformaldehyde and then blocked with 10% goat serum/PBS. Incubation with primary antibodies was performed overnight at 4 °C, followed by washing and incubation with PE-conjugated secondary antibodies. Primary antibodies used for immunostaining and immunohistochemistry included mouse anti-human K14 (Santa Cruz Biotechnology) monoclonal, mouse anti-human K10 (Santa Cruz Biotechnology) monoclonal, and mouse anti-human Involucrin (Santa Cruz Biotechnology) monoclonal antibodies. Finally, the samples were counterstained with DAPI (Southern Biotech, Birmingham, USA) and observed under a fluorescence microscope (Leica DM 2500). The artificial skin was fixed in 4% paraformaldehyde, dehydrated, and embedded in paraffin for haematoxylin-eosin staining.

### Statistical analysis

The data, which are presented as the mean ± SD, were obtained after performing at least 3 independent experiments and subsequently analysed using Prism 5 software (GraphPad Software, San Diego, CA, USA). The real-time PCR data are presented as the fold-change in gene expression normalized to GAPDH (used as a reference gene) and relative to the controls using the 2^−ΔΔCT^ method. Differences between the sham, fibroblast and hAFS groups were analysed using the *t*-test and considered statistically significant at P < 0.05.

## Additional Information

**How to cite this article**: Sun, Q. *et al.* Amniotic fluid stem cells provide considerable advantages in epidermal regeneration: B7H4 creates a moderate inflammation microenvironment to promote wound repair. *Sci. Rep.* **5**, 11560; doi: 10.1038/srep11560 (2015).

## Supplementary Material

Supplementary Information

## Figures and Tables

**Table 1:** RT-PCR primers.

**Gene Name**	**Amplicon length (bp)**	**Forward primer (5′-3′)**	**Reverse primer (5′-3′)**
hKRT14	516	AGATTCTCACAGCCACAGTGGACA	ACTGCAGCTCAATCTCCAGGTTCT
hKRT8	222	AGGCATCACCGCAGTTACG	TTGCTTCGAGCCGTCTTCT
hKRT18	268	CCGCTACGCCCTACAGAT	CACTTTGCCATCCACTATCC
hKRT19	461	AGGTGGATTCCGCTCCGGGC	ATCTTCCTGTCCCTCGAGCA
hβ1-integrin	260	AATGTTTCAGTGCAGAGCC	TTGGGATGATGTCGGGAC
hKRT5	851	AGGGCACCAAGACTGTGA	GACTGGTCCAACTCCTTCTC
hOct4	565	ATGTCAGGGCTCTTTGTCC	TGGCACGCACCTGTAATC
hSox2	215	TGGACAGTTACGCGCACAT	CGAGTAGGACATGCTGTAGGT
hKlf4	291	CCTGGGTCTTGAGGAAGTG	GCCTTGAGATGGGAACTCTT
hc-Myc	962	AGAAGAACCACGAAGAGGAA	ATACAAAGTGGACAAAGAGCC
hRex-1	327	ATGTCAGGGCTCTTTGTCC	AGCTGCTAAGTTCTGGGTTAA
hNanog	212	CTTGCCTTGCTTTGAAGCATCCGA	CTGCAGAAGTGGGTTGTTTGCCTT
hGAPDH	293	GAAACTGTGGCGTGATGG	GGGTGTCGCTGTTGAAGT

**Table 2:** Real-time PCR primers.

**Gene Name**	**Amplicon length (bp)**	**Forward primer (5′-3′)**	**Reverse primer (5′-3′)**
mbFGF	104	GCGACCCACACGTCAAACTA	CCGTCCATCTTCCTTCATAGC
hbFGF	82	AGAAGAGCGACCCTCACATCA	CGGTTAGCACACACTCCTTTG
mCXCL12	118	TGCATCAGTGACGGTAAACCA	CACAGTTTGGAGTGTTGAGGAT
hCXCL 12	88	ATTCTCAACACTCCAAACTGTGC	ACTTTAGCTTCGGGTCAATGC
mVEGF	233	AGAGCAACATCACCATGCAG	CAGTGAACGCTCCAGGATTT
hVEGF	75	AGGGCAGAATCATCACGAAGT	AGGGTCTCGATTGGATGGCA
mCXCR4	109	CCACGCCACCAACAGTCAGA	GGCAAAGATGAAGTCGGGAATA
hCXCR4	236	AACTTCCTATGCAAGGCAGT	TATCTGTCATCTGCCTCACT
mIL-6	88	TCTATACCACTTCACAAGTCGGA	GAATTGCCATTGCACAACTCTTT
hIL-6	149	ACTCACCTCTTCAGAACGAATTG	CCATCTTTGGAAGGTTCAGGTTG
mMac3	111	ATGTGCCTCTCTCCGGTTAAA	GCAAGTACCCTTTGAATCTGTCA
hMac3	184	GAAAATGCCACTTGCCTTTATGC	AGGAAAAGCCAGGTCCGAAC
mCox2	124	TGCACTATGGTTACAAAAGCTGG	TCAGGAAGCTCCTTATTTCCCTT
hCox2	90	ATGCTGACTATGGCTACAAAAG	TCGGGCAATCATCAGGCAC
mTGF-ß1	91	CCACCTGCAAGACCATCGAC	CTGGCGAGCCTTAGTTTGGAC
hTGF-ß1	86	CAATTCCTGGCGATACCTCAG	GCACAACTCCGGTGACATCAA
mKGF	84	ACCTGAGGATTGACAAACGAGG	CCACGGTCCTGATTTCCATGA
hKGF	123	TCCTGCCAACTTTGCTCTACA	CAGGGCTGGAACAGTTCACAT
mIL-1ß	116	GAAATGCCACCTTTTGACAGTG	TGGATGCTCTCATCAGGACAG
hIL-1ß	84	TTCGACACATGGGATAACGAGG	TTTTTGCTGTGAGTCCCGGAG
mTNF-α	122	CTGAACTTCGGGGTGATCGG	GGCTTGTCACTCGAATTTTGAGA
hTNF-α	91	GAGGCCAAGCCCTGGTATG	CGGGCCGATTGATCTCAGC
mGAPDH	85	ATCATCCCTGCATCCACT	ATCCACGACGGACACATT
hGAPDH	197	GGAGCGAGATCCCTCCAAAAT	GGCTGTTGTCATACTTCTCATGG
