# Supplementary material for: Amniotic fluid stem cells provide considerable advantages in epidermal regeneration: B7H4 creates a moderate inflammation microenvironment to promote wound repair
Source: Sci Rep. 2015 Jun 23;5:11560. doi: 10.1038/srep11560 (PMC4477371; doi:10.1038/srep11560)
Supplement: Supplementary Information [file srep11560-s1.pdf]

Amniotic fluid stem cells provide considerable advantages in epidermal regeneration: B7H4 creates a moderate inflammation microenvironment to promote wound repair

Qing Sun[1, +], Fang Li[1, +], Hong Li[2], Rui-Hua Chen[3], Yan-Zheng Gu[3], Ying Chen[2], Han-Si Liang[1], Xin-Ran You[1], Si-Si Ding[1], Ling Gao[3], Yun-Liang Wang[3], Ming-De Qin[1, *], Xue-Guang Zhang[1, 3, *]

[+]Authors contributed equally

[*]Co-corresponding authors

[1] The Stem Cell and Biomedical Material Key Laboratory of Jiangsu Province (the State Key Laboratory Incubation Base), Suzhou, Jiangsu Province, P.R. China

[2] Center for Reproduction and Genetics, Suzhou Hospital Affiliated to Nanjing Medical University, P.R. China

[3] Jiangsu Institute of Clinical Immunology, The First Affiliated Hospital of Soochow University, P.R. China

Correspondence:

Xue-Guang Zhang. PhD. The Stem Cell and Biomedical Material Key Laboratory of Jiangsu Province (the State Key Laboratory Incubation Base), Suzhou, Jiangsu Province, P.R. China. Jiangsu Institute of Clinical Immunology, The First Affiliated Hospital of

Soochow University, P.R. China, 708 RenMin Road, Soochow, P.R. China. Telephone: 86-512-65732002; E-mail: xueguangzh@126.com

Ming-de Qin. PhD. The Stem Cell and Biomedical Material Key Laboratory of Jiangsu Province (the State Key Laboratory Incubation Base), Suzhou, Jiangsu Province, P.R. China, 708 RenMin Road, Soochow, China. Telephone: 86-512-65125011; E-mail: leopardxq@hotmail.com

Supplementary Figures

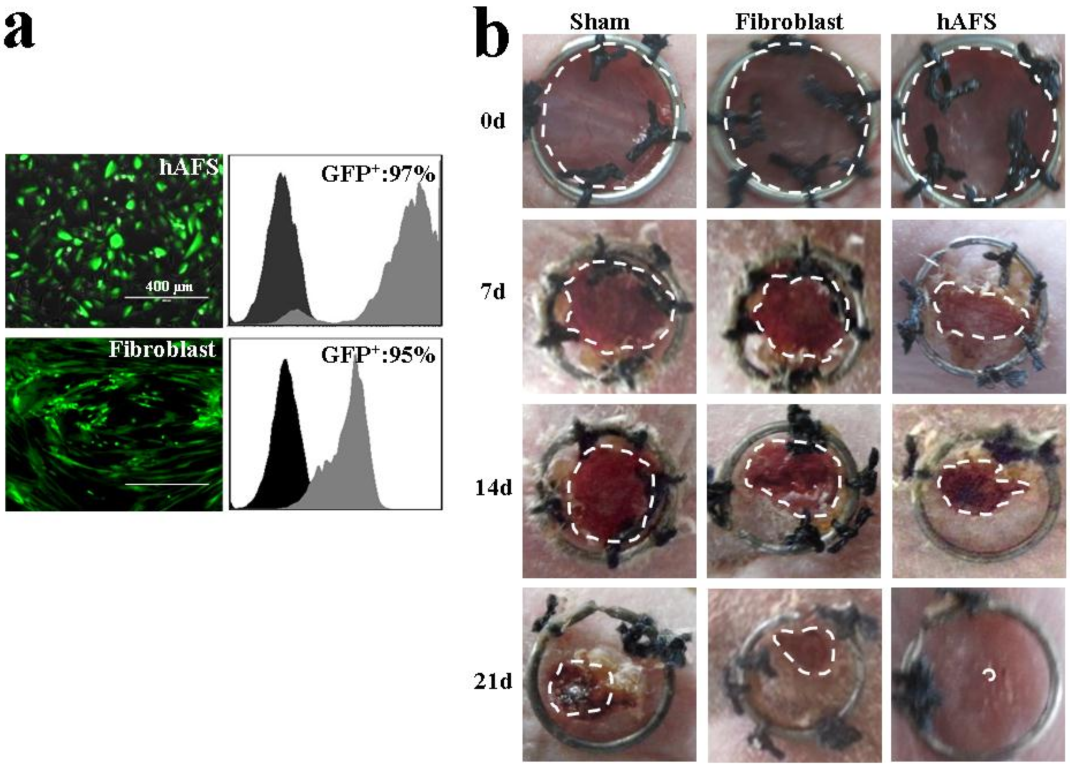

Supplementary Figure S1. Excisional wound model construction and GFP-positive hAFS cell transplantation

(a) A GFP reporter gene was delivered into hAFS cells and fibroblasts using a lentiviral vector. Immunofluorescence and flow cytometry show that almost 100% of hAFS cells and fibroblasts are GFP positive. (b) Original photographs of (Figure 4a, representative) the wounds with GFP-positive hAFS cells, fibroblasts or vehicle (sham) at days 7, 14 and 21 are shown. Over time, hAFS cell-treated wounds exhibited accelerated wound closure in BALB/c mice compared with mice treated with the fibroblast or sham groups. At 21 days, wounds in hAFS cell-treated mice achieved almost complete wound closure,

whereas no completely closed wounds were observed in the fibroblast- or sham-treated groups. The wounds are indicated by white circles.

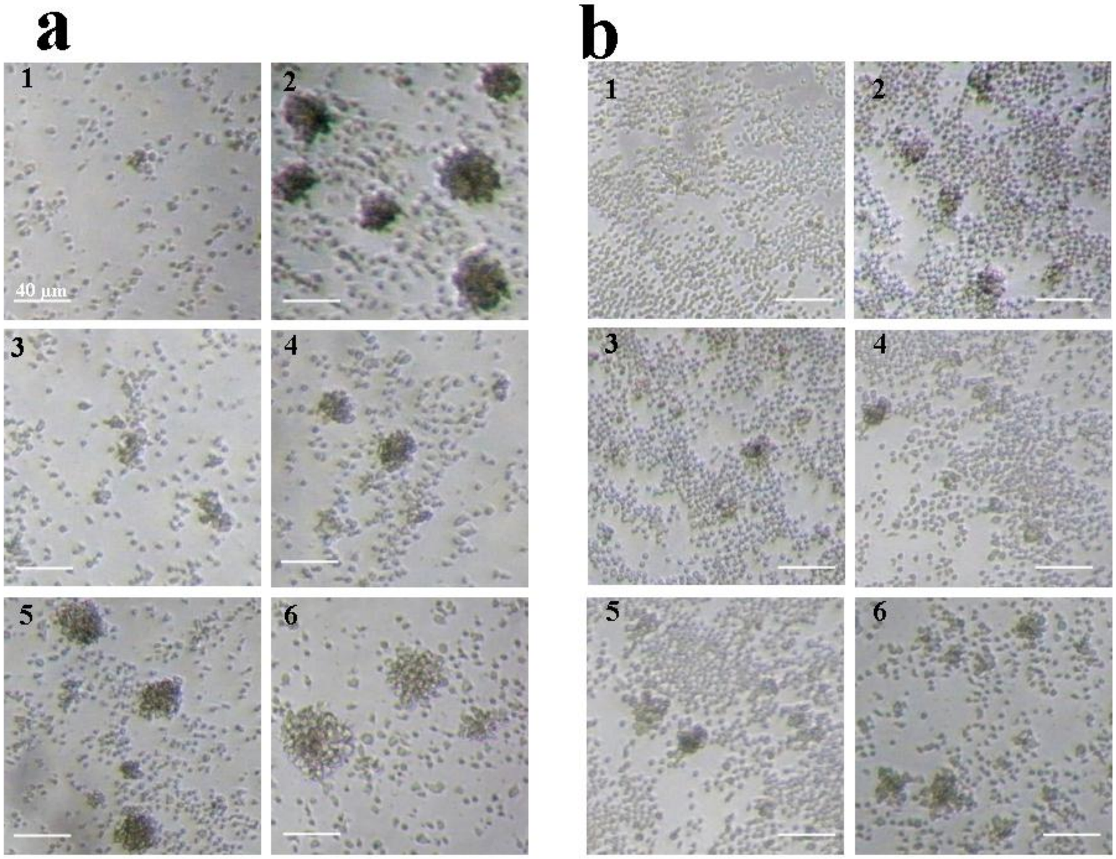

Supplementary Figure S2. The role of the B7H4 molecule in the proliferation of T lymphocytes

(a) Analysis of the inhibitory effect of hAFS cells on human lymphocytes. The corresponding cell images of Fig. 6a. 1: human T lymphocyte; 2: human T lymphocyte + CD3; 3: human T lymphocyte + CD3 + hAFS cells; 4: human T lymphocyte + CD3 + hAFS cells+ B7H1 blocking antibody; 5: human T lymphocyte + CD3 + hAFS cells + B7H4 blocking antibody; 6: human T lymphocyte + CD3 + hAFS cells + B7H1 blocking

antibody + B7H4 blocking antibody; 7: human T lymphocyte + CD3 + hAFS cells + mIgG. (b) Analysis of the inhibitory effect of hAFS cells on mouse lymphocytes. The corresponding cell images of Fig. 6b. 1: mouse T lymphocyte; 2: mouse T lymphocyte + CD3; 3: mouse T lymphocyte + CD3 + hAFS cells; 4: mouse T lymphocyte + CD3 + hAFS cells + B7H1 blocking antibody; 5: mouse T lymphocyte + CD3 + hAFS cells + B7H4 blocking antibody; 6: mouse T lymphocyte + CD3 + hAFS cells + B7H1 blocking antibody + B7H4 blocking antibody; 7: mouse T lymphocyte + CD3 + hAFS cells + mIgG.

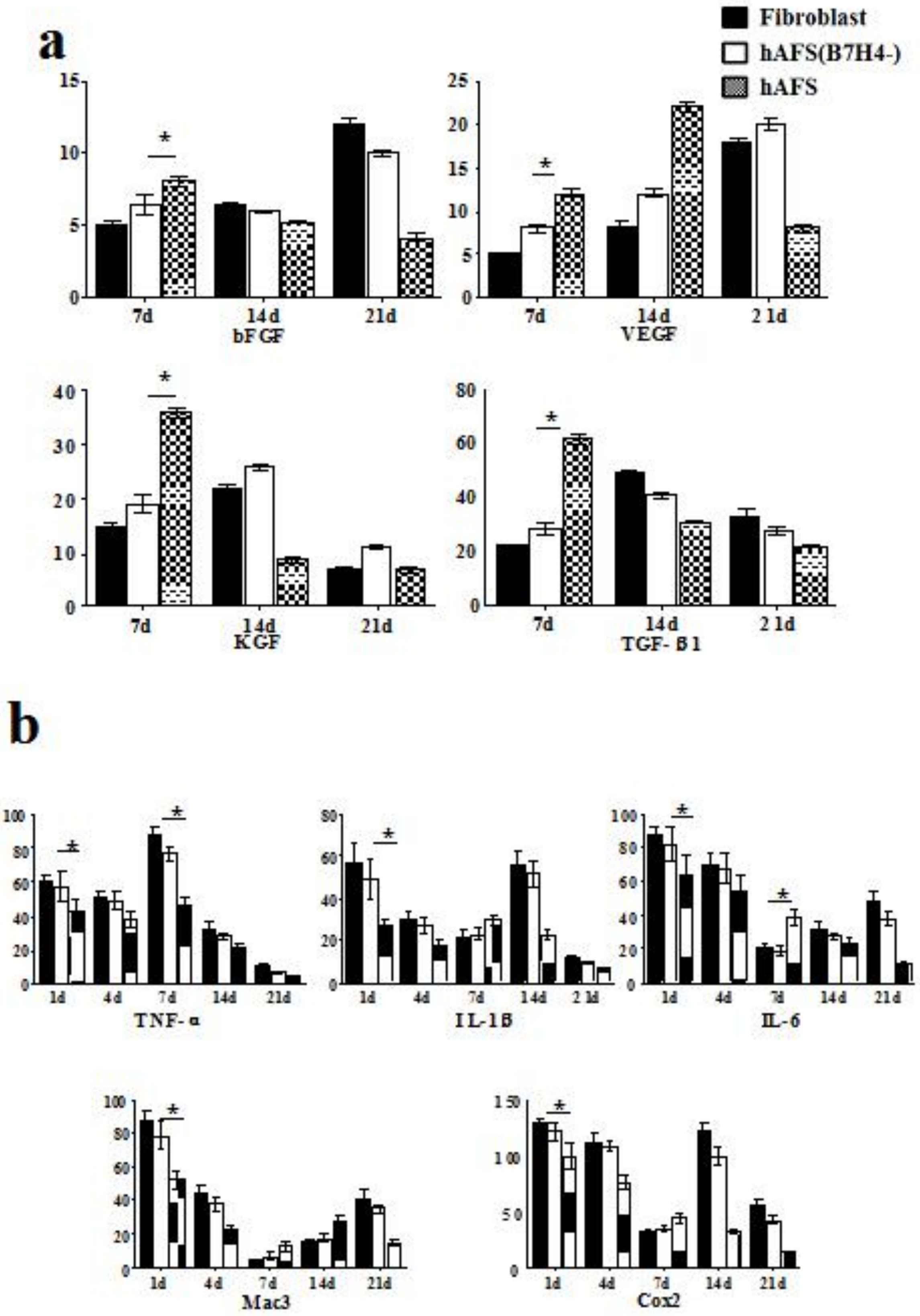
a
Fibroblast
hAFS(B7H4-)
hAFS
7d
14d
21d
bFGF
VEGF
KGF
TGF-β1
b
1d
4d
7d
14d
21d
TNF-α
IL-1β
IL-6
Mac3
Cox2

Supplementary Figure S3. B7H4 downregulated hAFS cells nullify the advantages of hAFS on accelerated healing and moderate inflammatory reactions during wound repair

(a) Real-time PCR analysis of mRNA expression of repair-related factors (bFGF, VEGF, TGF-β1, KGF) at days 7, 14, 21 in hAFS cell-, fibroblast- and B7H4-downregulated hAFS treatment groups. (b) Real-time PCR analysis of mRNA expression of inflammatory factors (TNF-α, Cox2, Mac3, IL-6, IL-1β) at days 1, 4, 7, 14 and 21 in hAFS cell-treated, fibroblast and B7H4-downregulated hAFS groups. The data are presented as the mean±SD of three independent experiments: analysis was performed with GraphPad Prism; *: $p<0.05$.

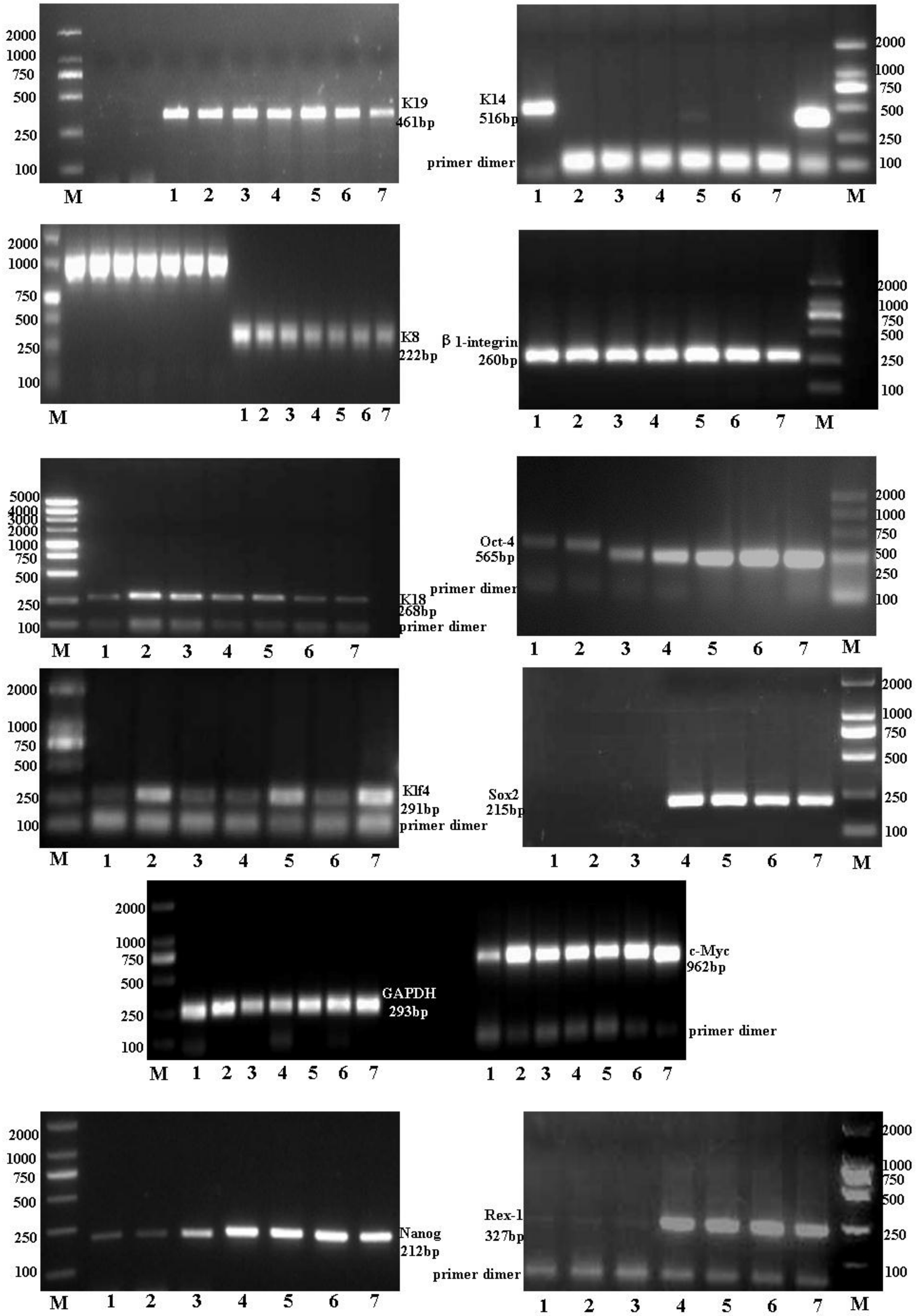
2000
1000
750
500
250
100
M
1 2 3 4 5 6 7
K19
461bp
K14
516bp
primer dimer
1 2 3 4 5 6 7 M
K8
222bp
β 1-integrin
260bp
5000
4000
3000
K18
268bp
primer dimer
Oct-4
565bp
Klf4
291bp
Sox2
215bp
GAPDH
293bp
c-Myc
962bp
Nanog
212bp
Rex-1
327bp

Supplementary Figure S4. Full-length images of RT-PCR in Figure 1a. RT-PCR assay for determining relative mRNA expression levels of several genes in keratinocytes, hAFS cells and hESCs. GAPDH was used as an internal loading control (1: keratinocyte; 2-6: three lines of hAFS cells with different passage numbers; 7: hESC. M: marker).

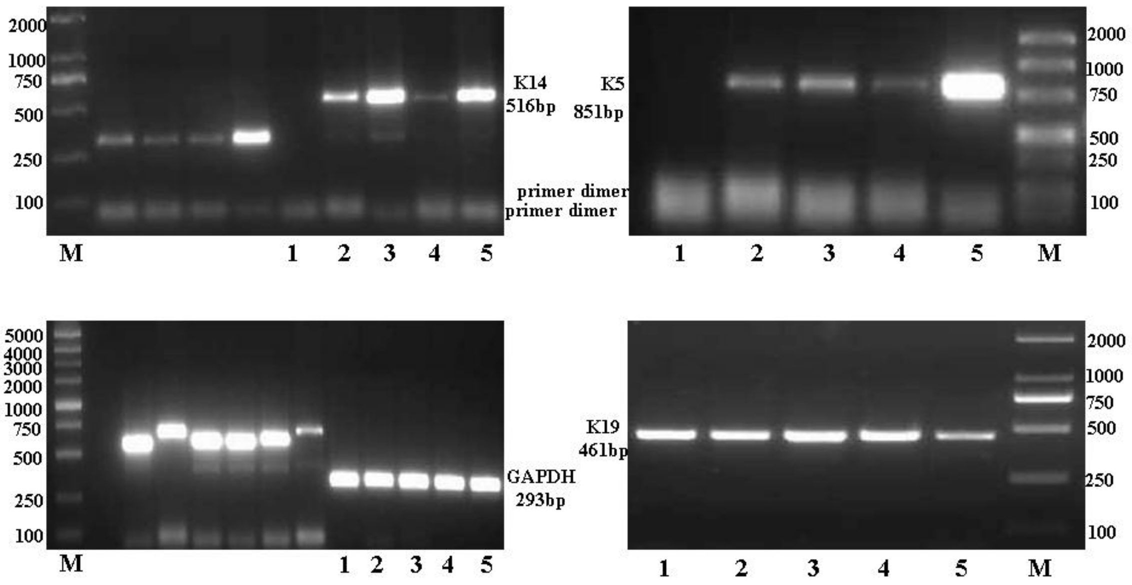

Supplementary Figure S5 Full-length images of RT-PCR in Figure 2b. RT-PCR assay for determining the relative mRNA expression level of several genes in hAFS cells, keratinocytes derived from hAFS cells (hAFS-K) and keratinocytes. GAPDH was used as an internal loading control (1: hAFS cells; 2-4: three lines of hAFS-K; 5: keratinocyte; M: marker).
